# Susceptibility Evaluation of Fall Armyworm (*Spodoptera frugiperda*) Infesting Maize in Kenya against a Range of Insecticides

**DOI:** 10.1155/2022/8007998

**Published:** 2022-08-08

**Authors:** Savinda Njeri Gichere, Kakai Shem Khakame, Okoth Patrick

**Affiliations:** ^1^Department of Biological Sciences, School of Natural and Applied Sciences, Masinde Muliro University of Science and Technology, P.O. Box 190-50100, Kakamega, Kenya; ^2^Department of Agricultural Resource Management, School of Agriculture, University of Embu, P.O. Box 6-60100, Embu, Kenya

## Abstract

The fall armyworm, *Spodoptera frugiperda* (J. E. Smith), is a worldwide pest of gramineous crops and a major pest of corn. Kenya has, in the recent years, reported massive outbreaks of this pest causing huge economic losses in maize fields. The indiscriminate use of insecticides has led to the evolution of insecticide resistance. This presents serious challenges to the control of pests including fall armyworm. The fall armyworm infestation has greatly threatened food security in Kenya. Consequently, this has heightened the need to evaluate the susceptibility of the fall armyworm to commonly used insecticides in Kenya. In this study, thirteen populations of the fall armyworm were sampled from thirteen counties of Kenya and determined its susceptibility to a range of insecticides using leaf-dip bioassay method. The current study illustrated the high toxicity of spinetoram, spinosad, lufenuron, and pyridaben to fall armyworm while indoxacarb, deltamethrin, lambda-cyhalothrin, imidacloprid, and abamectin exhibited relatively low toxicity to fall armyworm. Possible cross-resistance between abamectin, imidacloprid, deltamethrin, indoxacarb, spinosad, spinetoram, and lufenuron was determined through pair-wise correlational analyses. Results of this study revealed no cross-resistance between lambda-cyhalothrin with all other insecticides tested. Susceptibility monitoring of the fall armyworm can be a valuable strategy in the control of fall armyworm in the field populations. This can help inform the policy to design management strategies that promote the judicious use of these chemicals and prolong their efficacy in the management of the fall armyworm in Kenya.

## 1. Introduction

The fall armyworm, *Spodoptera frugiperda* (J. E. Smith), originated from the tropical and subtropical regions of America and is a migratory polyphagous pest [1]. The species has a wide host range with over eighty crops which include cereals, vegetable crops, sugarcane, and pulses [2]. This pest was identified in West Africa in 2016 and has since spread to East African countries including Kenya [3]. In March 2017, the pest was first reported in Western Kenya in the counties of Busia, Trans-Nzoia, Bungoma, Nandi, and Uasin-Gishu [4]. It was suggested that this pest could have spread to Africa either through direct flight, cargo containers, or airplane holds [5, 6].

In most parts of Sub-Saharan Africa, maize is a staple food. According to Sisay et al. [7], the annual maize consumption in Zimbabwe, Zambia, and Malawi is 153, 168, and 181 kg, respectively. In South Africa, Malawi, and Zambia, the daily mean consumption of maize is 252.7 g per person [8]. Maize is grown in six agroecological zones in Kenya [9, 10]. The zones include western, central, and highland tropics with an average production of 2.5 t/ha. The low land zones include the coastal and lake basin regions with an average production of 1.5 t/ha.

The fall armyworm (FAW) infestation and damage reduce the maize yield and negatively affect the national GDP due to diminished market access [11, 12]. All growth stages of various crops are affected by the pest. Its damage to maize includes defoliation and ear damage which result in low quality and reduced yields [12]. According to Sisay et al. [7], the pest has positively been identified in 44 countries in Africa. The FAW has the potential of causing enormous losses approximated at US$ 2.4–6.2 billion when left uncontrolled [5]. A study by Kumela et al. [13] reported that farmers from Ethiopia and Kenya estimated maize infestation by the FAW on a range of 24.1% to 39.4% and 38% to 53.9%, respectively. It is also estimated in the two countries that the pest can cause a yield reduction of 934 kg/ha and 1381 kg/ha, respectively.

In Latin American countries, FAW mostly affects maize more than cotton, directly affecting crop productivity by damaging the floral parts of the crop and has the potential to reach 100% losses in some tropical areas if mitigation measures are not put in place [14, 15].

Insecticides have been incorporated into the integrated pest management (IPM) recommendations to manage the FAW due to its ability to feed on a broad host range and migrate long distances rendering other control options ineffective. Over-dependence on chemical insecticides has led to the development of insecticide resistance by this pest to most classes of insecticides [16]. Carvalho et al. [17] demonstrated that the pest had developed resistance to organophosphates and pyrethroids. Resistance has also been reported in carbamates, organophosphates, pyrethroids, and *Bacillus thuringiensis* [18]. It is against this background that investigating emerging resistance development, determining baseline susceptibility, and evaluating cross-resistance will help develop valuable control options to effectively manage the FAW infestation in Kenya.

## 2. Materials and Methods

### 2.1. Study Site

**Figure 1:**
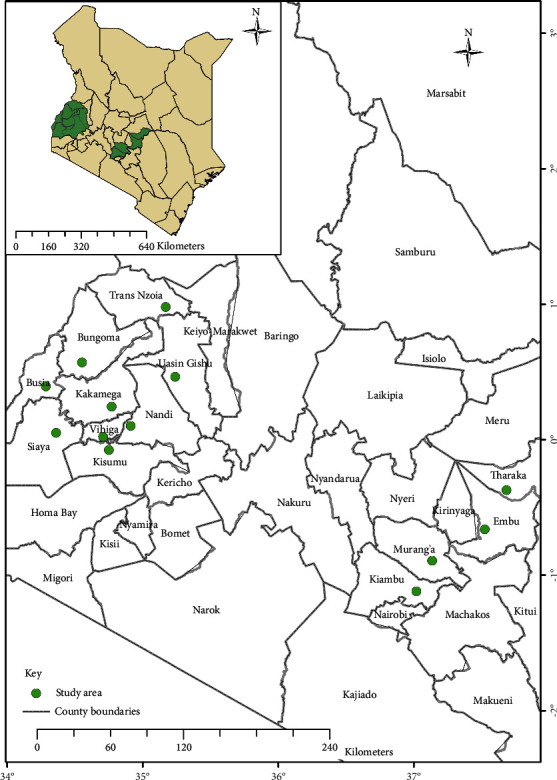
Sampling sites of the field populations of *S. frugiperda* from Kenya. The green dots represent sampling locations (Table 1).

The sampling area consisted of the five former provinces of Kenya (Western, Central, Eastern, Nyanza, and Rift Valley). Within these provinces, different counties were sampled which included Kakamega, Busia, Kisumu, Trans-Nzoia, Uasin-Gishu, Siaya, Vihiga, Embu, Tharaka-Nithi, Nandi, Kiambu, Muranga, and Bungoma counties as shown in Table 1 and Figure 1. These regions were of great importance to this study as they form most of the Kenyan main producing areas of maize. Most farmers in these regions indiscriminately use the registered chemicals and the Kenyan authorities have no definite program for their use because there is no published data on the use of chemicals in Kenya.

### 2.2. Insects

*Spodoptera frugiperda* reference strain was raised for four years without exposure to any chemical insecticide by Kalmer Agricultural consultants. Thirteen populations of the FAW were sampled from thirteen countries of Kenya (Figure 1 and Table 1). 200 fourth to sixth-instar larvae of *Spodoptera frugiperda* were sampled in selected fields in a semisystematic manner using a “W” pattern used in scouting [19]. The larvae were placed in 10 ml plastic jars containing soft maize leaves as a diet. They were visually assessed and confirmed as the FAW using morphological features. Insect rearing was conducted as per Moreno et al. [20] with some modifications. The larvae were held at room temperature, in plastic cans for further development. The plastic cans contained a moisturized kitchen towel and soft maize plant leaves as a diet. The lid contained a muslin cloth for aeration and the developmental stage of the insect was checked every day while pupated larvae were collected and transferred to different small plastic containers. Forty pupae were placed in insect cages after turning their color from orange-red to dark red for adult emergence and mating and this was done separately for each field population. The cages were covered internally with grease paper for oviposition. The adults were fed on 10% honey solution which was replaced after two days. After the third day of adult emergence, the moths from each cage were transferred to 1-liter plastic containers with lids covered internally with a muslin cloth and 10% honey solution in the cotton roll to be used as food. Egg masses were placed in new plastic containers with moistened kitchen towels and soft maize plants and held at 26 ± 1°C until hatching. Bioassays were carried out with the 3^rd^ instar F2 larvae.

### 2.3. Insecticides

Commercially formulated insecticides used in this study are summarized in Table 2 and included abamectin 18 g/L (Deacarid 1.8 EC, Bio-Medica Laboratories, Nairobi, Kenya), pyridaben 200 g/L (Genomite 200 EC, Geneva Agrochemicals Ltd., Thika, Kenya), lufenuron 50 g/L (Match 050 EC, Syngenta East Africa Ltd., Nairobi, Kenya), imidacloprid 200 g/L (Concord 20 SL, Agri Scope (Africa) Ltd., Nairobi, Kenya), deltamethrin 25 g/L (Katrin 25 EC, Twiga Chemicals Industries Ltd., Nairobi, Kenya), lambda-cyhalothrin 17.5 g/L (Duduthrin 1.75 EC, Twiga Chemicals Industries Ltd., Nairobi, Kenya), spinosad 480 g/L (Tracer 480 SC, Dow Chemical East Africa Ltd., Nairobi, Kenya), spinetoram 120 g/L (Radiant 120 SC, Dow Chemical East Africa Ltd., Nairobi), and indoxacarb 150 g/L (Avaunt 150 EC, Elgon Kenya Ltd., Nairobi Kenya).

### 2.4. Bioassays

The third-instar larvae from the F2 generation were exposed to varying insecticide concentrations using the leaf-dip bioassay (IRAC method No. 7) [21]. Five serial dilutions for each insecticide were prepared with distilled water with their concentrations calculated in mg/L. Since toxicity is related to the logarithm of dose, different dose ranges in geometric series were preferred for each test insecticide covering 5% to 100% mortality. Cleansed maize leaves (the youngest leaf, from 1 to 3 cm of the leaf apex) were excised into leaf discs of 5 cm in diameter and dipped in the insecticide solutions for 5 seconds with gentle agitation. The leaf discs were allowed to surface dry on paper toweling for an hour. Distilled water was used as a control. The Petri dishes were lined up with moistened kitchen towels to avoid desiccation and the leaf discs were placed individually in them. Water was added daily to keep the kitchen towel moist so that leaf turgor is maintained. Ten third-instar larvae were introduced into each Petri dish and replicated four times. The larvae mortality was recorded after 48 h for rapidly acting insecticides and 72 h for the slow-acting insecticides. The larvae were considered dead if unable to move when probed with a horse brush. The bioassay was performed at an average temperature and relative humidity of 26 ± 1°C and 60–70%, respectively.

### 2.5. Statistical Analysis

The PoloPlus program [22] was used for probit analysis. Overlapping of the 95% fiducial limits was used as the criteria to determine significant differences in response among the insecticides used in the bioassays [23]. The LC_50_ values of the field populations were divided by the LC_50_ value of the susceptible strain to obtain the resistance ratios, while the LC_50_ value of the least toxic compound was divided by the LC_50_ value of the most toxic compound to obtain relative potency ratios to estimate the potency of the active ingredients [20]. Pairwise correlation coefficients were evaluated among the log LC_50_ values in the field-collected populations and tested chemicals by use of the analysis of Pearson's correlation using the IBM SPSS Statistics software package (Version 22.0) to assess the possible cross-resistance among different chemicals [24]. A *p* value of less than 0.05 was regarded as statistically significant [24].

## 3. Results

All the 13 FAW field populations tested against lambda-cyhalothrin exhibited low levels of resistance (3-4-fold) compared to the reference strain. The LC_50_ values were 139.63 mg/L for the KS (Kisumu population) and 197.359 mg/L for the TZ (Trans- Nzoia population), showing a 1.4-fold difference. The LC_50_ values of all the FAW populations had overlapping 95% fiducial limits indicating that their response to lambda-cyhalothrin was not significantly different (Table 3).

The LC_50_ values of deltamethrin against the FAW were 130.604 mg/L for TZ (Trans-Nzoia) and 61.267 mg/L for Siaya populations. The susceptible strain exhibited an LC_50_ value of 29.463 mg/L (Table 3). The fiducial limits obtained at this LC_50_ value overlapped among all the strains, suggesting similar toxicity of deltamethrin across all the populations which were also observed in the reference strain. The resistance ratios ranged from 2- to 4-fold, an indication of low levels of resistance.

The *S. frugiperda* population from TZ had the highest LC_50_ value of 26.284 mg/L for indoxacarb. The SA population recorded the lowest LC_50_ value of 14.206 mg/L (Table 3). The populations exhibited very low resistance (1-2-fold) to indoxacarb.

The LC_50_ values for pyridaben varied from 6.955 mg/L (TZ) to 5.328 mg/L (VH), showing a 1.3-fold difference (Table 4). The low LC_50_ values indicate that pyridaben is a highly potent active ingredient against the FAW. The populations had the same resistance ratio of 1. This demonstrates that the field populations of FAW in Kenya are still very susceptible to this chemical. The slope values obtained were similar indicating the heterogeneity of these populations in their response to pyridaben. The LC_50_ values of abamectin were higher (Table 4) compared to other active ingredients used in this study. These results suggest that this compound is less effective in controlling FAW from the sampled regions. This implies that large quantities of the insecticide are needed in the control of FAW making it less cost-effective.

As evidenced by similar values of the slopes and the overlapping 95% fiducial limits, all the populations had a similar response to the imidacloprid toxicity. The LC_50_ values of imidacloprid against the field populations ranged from 1168.392 mg/L for the SA population to 1748.019 mg/L for the TZ population (Table 4). The high LC_50_ values demonstrate that the field populations of FAW are less susceptible to imidacloprid and thus less potent in its management. The results displayed low levels of resistance to this active ingredient as the resistance ratios ranged from 1 to 2-fold.

The LC_50_ values for spinosad against the FAW varied between 0.757 mg/L and 0.363 mg/L (Table 5), showing 2.09-fold variability between the least and most sensitive populations was observed. The TZ population exhibited a (3-fold) resistance ratio compared to other field populations. The log dose-probit regression slopes for spinosad among the populations were similar to the indication of similar levels of toxicity to the populations.

The LC_50_ values of spinetoram ranged from 0.479 mg/L for the TZ population to 0.158 mg/L for the KS population, as shown in (Table 5). In this study, spinetoram was the most toxic insecticide and all populations displayed very low levels of resistance (1 to 3 fold) to this chemical. Lufenuron exhibited the LC_50_ values against the UG population (6.132 mg/L) and the SA population (4.227 mg/L) showing 1.5-fold difference (Table 5). All populations had a resistance ratio of 2-fold, except the SA population which had a resistance ratio of 1. Populations from the Rift valley region displayed high LC_50_ values compared to populations in other regions suggesting that these three populations were less susceptible to lufenuron.

### 3.1. Relative Potency of the Nine Active Ingredients

**Figure 2:**
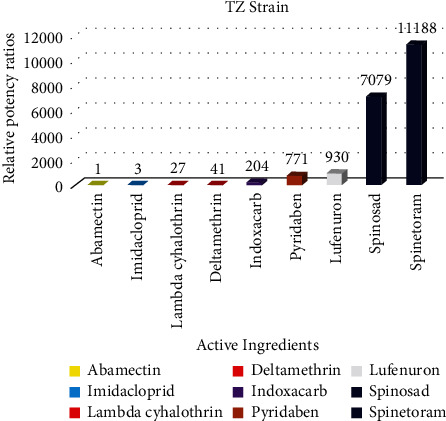
Relative potency ratios of different insecticides against fall armyworm in Kenya.

Spinosyns were the most potent insecticides against all the populations of the FAW. However, spinetoram had a relative potency ratio of 11188 against spinosad with a relative potency ratio of 7079 (Figure 2). This indicated that the FAW is highly susceptible to these two newer chemistries. All insecticides had a relative potency ratio of above 20, except for abamectin (1) and imidacloprid (3). The abamectin having the lowest relative potency ratio was used as the index insecticide in the calculation of relative potency ratios. The findings show that abamectin is the least efficacious compound, hence the field populations are less susceptible to this insecticide. Pyridaben and lufenuron had relatively high ratios, hence they are also relatively effective against the field populations. This is also evidenced by the lower LC_50_ values from the susceptibility bioassay results.

The assessment of pairwise correlation coefficients was done between the log LC_50_ values of the tested chemicals for the *Spodoptera frugiperda* field-collected populations (Table 6). Consequently, resistance to lufenuron had a significant correlation with pyridaben resistance (*r* = 0.878, *p* < 0.01), abamectin (*r* = 0.976, *p* < 0.01), imidacloprid (*r* = 0.907, *p* < 0.01), deltamethrin (*r* = 0.959, *p* < 0.01), indoxacarb (*r* = 0.944, *p* < 0.01), spinosad (*r* = 0.912, *p* < 0.01), and spinetoram (*r* = 0.818, *p* < 0.01). The significant correlation was also observed in spinetoram with pyridaben resistance (*r* = 0.943, *p* < 0.01), abamectin (*r* = 0.840, *p* < 0.01), imidacloprid (*r* = 0.848, *p* < 0.01), deltamethrin (*r* = 0.890, *p* < 0.01), indoxacarb (*r* = 0.939, *p* < 0.01), and spinosad (*r* = 0.922, *p* < 0.01). Similarly, spinosad had positive significant correlations with pyridaben (*r* = 0.907, *p* < 0.01), abamectin (*r* = 0.936, *p* < 0.01), imidacloprid (*r* = 0.854, *p* < 0.01), deltamethrin (*r* = 0.935, *p* < 0.01), and indoxacarb (*r* = 0.947, *p* < 0.01). The indoxacarb had significant correlation with pyridaben (*r* = 0.973, *p* < 0.01), abamectin (*r* = 0.952, *p* < 0.01), imidacloprid (*r* = 0.949, *p* < 0.01), and deltamethrin (*r* = 0.973, *p* < 0.01). Significant correlation was also observed in deltamethrin with pyridaben (*r* = 0.940, *p* < 0.01), abamectin *r* = 0.982, *p* < 0.01), and imidacloprid (*r* = 0.931, *p* < 0.01). The imidacloprid exhibited a strong correlation with pyridaben (*r* = 0.951, *p* < 0.01) and abamectin (*r* = 0.923, *p* < 0.01). The abamectin exhibited significant correlation with pyridaben (*r* = 0.907, *p* < 0.01). However, there was no significant correlation of lambda-cyhalothrin with other eight tested chemicals in the collected *Spodoptera frugiperda* field populations.

## 4. Discussion

The current study evaluated the susceptibility of the FAW from Kenya to nine different insecticides with the different modes of action. These chemicals were deltamethrin, lambda-cyhalothrin, abamectin, spinosad, spinetoram, lufenuron, pyridaben, imidacloprid, and indoxacarb. These insecticides are readily available in the Kenyan market and the farmers have been using them to control different pests including the FAW indiscriminately. The findings of this study demonstrate an initial effort in developing susceptibility data for insecticides used to control the FAW in Kenya. In addition, the results obtained suggest that the pattern of the response of FAW for each of the nine insecticides used was similar across all sampled locations.

Pyrethroids target the voltage-gated sodium channels. They act by inhibiting channel deactivation and inactivation, thus stabilizing their open state, leading to prolonged channel opening [25]. The pyrethroids are a vital group used in the control of FAW in Mexico [21]. In the current study, FAW were less susceptible to lambda-cyhalothrin as compared to deltamethrin. For lambda-cyhalothrin, all the field populations had a significantly different response from the SUS strain based on the nonoverlapping 95% fiducial limits. A study by Kulye et al. [26] reported a lack of effectiveness of deltamethrin in the *S. frugiperda* populations. In the deltamethrin tested strains, only VH, BS, and SA had comparable susceptibility to the SUS strain (Table 3). Even though the resistance ratios for the two insecticides were very low, the high LC_50_ values obtained for these two pyrethroids suggest that they may no longer be effective in the management of the field populations of FAW. Our finding is in concurrence with the studies by Zhao et al. [23].

Indoxacarb binds and blocks sodium channels leading to pseudoparalysis [23]. The present study revealed high LC_50_ values of indoxacarb hence low potency against the pest. Nevertheless, the resistance of the pest populations to this compound was found to be very low (1- to 2-fold). Low variation in the susceptibility (4.6-fold) to indoxacarb was reported by Kaiser et al. [27]. Deshmukh et al. [28] reported low potency (2-fold) of indoxacarb against FAW collected from unsprayed maize farms in India using a similar bioassay method. Ahmad et al. [29] documented very low resistance of *Spodoptera exigua* to indoxacarb. In contrast, a moderate to a very high level of resistance of *Spodoptera exigua* from Pakistan and diamondback moth, *Plutella xylostella* (L.), from China to indoxacarb has been documented [30, 31].

From our findings, imidacloprid was the second least potent active ingredient from abamectin, with high LC_50_ values. These results suggest that large quantities of imidacloprid are needed to kill half of the FAW population. However, the resistance ratio was 1- to 2-fold. Previous studies have documented that various species including tobacco whitefly, small brown plant hopper, western flower thrips, and peach aphid have developed resistance to imidacloprid [32]. Our studies found that one of our *S. frugiperda* populations (KK) with an LC_50_ value of 4246 mg/L had a resistance ratio of 1.37-fold to abamectin. There are limited research findings on the efficacy of abamectin against FAW because it is largely used to control mites. Its derivative emamectin benzoate is usually effective against lepidopteran pests. A study by Zhao showed that emamectin benzoate had 2340-fold against the *Spodoptera frugiperda* with an LC_50_ value of 678 mg/L.

Spinosyns are allosteric modulators of nicotinic acetylcholine receptors and consist of two active ingredients, spinetoram and spinosad [33]. This group of insecticides has been a key component in the management of FAW [34]. Spinetoram is effective and has been used to control FAW in the field [34]. The current study showed that spinetoram has high toxicity against the fall armyworm. A study by Zhao et al. [23] using diet incorporation bioassay detected high toxicity of spinetoram against the FAW. Experiments by Hardke et al. [35] demonstrated that spinetoram was the most toxic of the insecticides tested against this pest with lower LC_50_ values than the values from the current study. Spinosad is also an essential chemical for controlling the FAW in Puerto Rico [20]. In this study, spinosad was second in terms of efficacy evidenced by the low LC_50_ values. Spinosad and spinetoram populations exhibited low resistance (1- to 3-fold). Pakistani populations of *Spodoptera litura* tested from 1997 to 2013 exhibited low resistance to spinosad [36]. On the contrary, Moreno et al. [20] reported a moderate resistance ratio (8-fold) to a Puerto Rico population to spinosad suggesting that the FAW populations from Kenya are more susceptible to this active ingredient.

Lufenuron is a benzoylurea that binds chitin synthase 1 in terrestrial arthropods resulting in inhibition of chitin biosynthesis [37]. Despite the different bioassay method used by Zhao et al. [23], lufenuron exhibited similar levels of toxicity in regard to our study. The current study revealed very low tolerance (1-2-fold) to this chemical.

The study of evaluating the susceptibility of the FAW populations to insecticides will help reduce the development of resistance and guide rotational programs in the field [23], through a strategy of multiple attacks where two or more unrelated insecticides are used. A negative correlation was reported by Zhao et al. [23] between spinetoram and lambda-cyhalothrin (*R* = −0.559). The current study revealed a weak correlation between the two chemicals (Table 6). Lambda-cyhalothrin and indoxacarb did not show any correlation [23]. We report no correlation between the two chemicals, an indication of a lack of cross-resistance. Muraro et al. [38] observed low levels of cross-resistance of abamectin to lambda-cyhalothrin, indoxacarb, and spinetoram. Our study revealed a strong correlation of abamectin to indoxacarb and spinetoram, but a weak correlation to lambda-cyhalothrin (Table 6). Lira et al. [34] reported the existence of cross-resistance between spinosad and spinetoram which can jeopardize their excellent efficacy against the *Spodoptera frugiperda* in the field. A recent study by Stacke et al. [39] reported that lambda-resistant strain showed low cross-resistance to deltamethrin (6.2-fold) in soybean looper, *Chrysodeixis includens* (Lepidoptera: Noctuidae). Our study revealed a weak correlation (*R* = 0.445) between deltamethrin and lambda-cyhalothrin.

In our current research, analysis of pairwise correlation of log LC_50_ values found levels of cross-resistance among the eight insecticides tested. However, lambda-cyhalothrin exhibited weak correlations to the eight insecticides tested implying a lack of cross-resistance to them.

## 5. Conclusions

This study reports the initial efforts to evaluate the susceptibility of the fall armyworm field populations from different agroecological regions of Kenya to various synthetic insecticides currently available on the Kenyan market. The data generated will be useful in informing policy and designing strategies for the management of fall armyworm in Kenya. The weak correlation exhibited by lambda-cyhalothrin to other eight insecticides can be exploited to develop rotational programs to preserve the efficacy of these chemicals in the field. An integrated approach to pest management will be required to manage the fall armyworm in the field.

## Figures and Tables

**Table 1:** Sampling site, date of sampling, and developmental stage of *Spodoptera frugiperda*.

Strain	Location	Site	Date of collection	Developmental stage
KK	Kakamega	0.28°N, 34.77°E	May 2021	4th and 6th instar larvae
VH	Vihiga	0.05°N, 34.72°E	May 2021	4th and 6th instar larvae
BS	Busia	0.46°N, 34.26°E	May 2021	4th and 6th instar larvae
BN	Bungoma	0.57°N, 34.55°E	June 2021	4th and 6th instar larvae
MR	Muranga	0.80°S, 37.96°E	May 2021	4th instar larvae
KA	Kiambu	1.12°S, 37.02°E	May 2021	4th and 6th instar larvae
EB	Embu	0.54°S, 34.46°E	June 2021	6th instar larvae
TN	Tharaka-Nithi	0.29°S, 37.72°E	June 2021	6th instar larvae
KS	Kisumu	0.04°S, 34.75°E	May 2021	4th and 6th instar larvae
SA	Siaya	0.05°N, 34.36°E	June 2021	4th and 6th instar larvae
UG	Uasin-Gishu	0.51°N, 35.24°E	June 2021	6th instar larvae
TZ	Trans-Nzoia	1.09°N, 35.62°E	June 2021	6th instar larvae
NN	Nandi	0.13°N, 34.88°E	June 2021	6th instar larvae

**Table 2:** Commercial insecticides applied against field-collected populations of *S. frugiperda*.

Commercial insecticide	Mode of action (IRAC)
Abamectin	Stimulates the gamma-aminobutyric acid (GABA) system
Spinosad	nAChR 4 allosteric modulator
Indoxacarb	Blocks the sodium channel
Imidacloprid	Blocks neurotransmission by postsynaptic antagonism of acetylcholine receptors
Deltamethrin	Sodium channel modulator
Lufenuron	Interferes with chitin synthesis polymerization and deposition
Lambda-cyhalothrin	Sodium channel modulator
Pyridaben	Mitochondrial complex 1 electron transport inhibitor
Spinetoram	nAChR 4 allosteric modulator

**Table 3:** Susceptibility of *Spodoptera frugiperda* field populations to lambda-cyhalothrin, deltamethrin, and indoxacarb.

Region	Insecticide	*P*	*N*	Slope ± SE	*χ * ^2^ (d*f*)	LC_50_ (mg/L)	95% FL (mg/L)	RR
Western	Lambda-cyhalothrin	SUS	200	2.06 ± 0.34	1.55 (3)	53.77	34.34–71.63	1
		KK	200	3.09 ± 0.77	0.68 (3)	143.37	104.41–170.45	2.67
		VH	200	3.53 ± 0.82	1.02 (3)	141.14	107.54–164.79	2.62
		BS	200	2.12 ± 0.41	0.68 (3)	147.45	113.38–187.44	2.74
		BN	200	2.07 ± 0.56	0.13 (3)	143.48	100.12–204.60	2.67
Central		MR	200	3.41 ± 0.78	0.81 (3)	165.77	133.51–195.41	3.08
		KA	200	3.72 ± 0.84	0.99 (3)	160	128.36–186.43	2.98
Eastern		EB	200	4.04 ± 0.80	1.31 (3)	153.73	126.04–177.29	2.86
		TN	200	3.92 ± 0.79	0.74 (3)	155.02	127.45–178.74	2.88
Nyanza		KS	200	4.45 ± 0.76	1.53 (3)	139.63	117.32–158.61	2.6
		SA	200	3.60 ± 0.89	1.67 (3)	140.62	102.98–165.58	2.62
Rift valley		UG	200	3.78 ± 0.79	0.57 (3)	179.13	152.25–208.14	3.33
		TZ	200	3.29 ± 0.82	2.64 (3)	197.36	165.03–241.37	3.67
		NN	200	3.61 ± 0.72	0.20 (3)	162.26	134.88–188.64	3.02

Western	Deltamethrin	SUS	200	1.59 ± 0.27	0.34 (3)	29.46	18.25–40.92	1
		KK	200	1.41 ± 0.27	0.15 (3)	64.71	40.96–95.51	2.2
		VH	200	1.46 ± 0.27	0.49 (3)	62.84	40.62–91.07	2.13
		BS	200	1.27 ± 0.26	2.25 (3)	66.31	40.83–101.65	2.25
		BN	200	1.99 ± 0.28	0.96 (3)	63.25	48.48–81.19	2.15
Central		MR	200	1.12 ± 0.25	0.34 (3)	100.82	62.83–179.24	3.42
		KA	200	1.36 ± 0.27	1.55 (3)	97.83	64.08–154.50	3.32
Eastern		EB	200	1.33 ± 0.26	0.35 (3)	73.34	47.39–110.38	2.49
		TN	200	1.11 ± 0.23	1.29 (3)	73.17	47.09–115.73	2.48
Nyanza		KS	200	1.50 ± 0.25	0.19 (3)	61.78	43.44–85.34	2.1
		SA	200	1.51 ± 0.27	1.03 (3)	61.27	40.19–87.49	2.08
Rift valley		UG	200	1.04 ± 0.27	0.23 (3)	122.95	69.82–264.10	4.17
		TZ	200	1.16 ± 0.26	0.30 (3)	130.6	82.17–248.80	4.43
		NN	200	1.46 ± 0.28	0.70 (3)	119.21	81.95–186.57	4.05

Western	Indoxacarb	SUS	200	1.95 ± 0.35	1.74 (3)	10.85	6.66–14.68	1
		KK	200	1.91 ± 0.37	2.06 (3)	16.03	10.72–21.05	1.48
		VH	200	1.86 ± 0.33	2.72 (3)	15.49	10.90–20.02	1.43
		BS	200	2.24 ± 0.45	1.58 (3)	16.47	10.66–21.41	1.52
		BN	200	1.81 ± 0.33	1.47 (3)	16.3	11.55–21.15	1.5
Central		MR	200	1.95 ± 0.37	0.51 (3)	21.92	16.31–28.21	2.02
		KA	200	1.98 ± 0.40	0.23 (3)	20.24	14.24–26.25	1.87
Eastern		EB	200	2.44 ± 0.52	0.73 (2)	18.66	12.33–23.74	1.72
		TN	200	1.92 ± 0.40	2.54 (3)	18.81	12.59–24.72	1.73
Nyanza		KS	200	1.62 ± 0.32	0.21 (3)	14.96	9.56–20.31	1.38
		SA	200	1.47 ± 0.32	1.05 (3)	14.21	8.47–19.84	1.31
Rift valley		UG	200	1.78 ± 0.40	0.13 (3)	25.94	18.54–35.57	2.39
		TZ	200	1.77 ± 0.39	2.53 (3)	26.28	19.04–35.81	2.42
		NN	200	1.71 ± 0.35	1.97 (3)	22.33	16.06–29.93	2.06

**Table 4:** Susceptibility of field populations of *Spodoptera frugiperda* to pyridaben, abamectin, and imidacloprid.

Region	Insecticides	*P*	*N*	Slope ± SE	*χ * ^2^ (d*f*)	LC_50_ (mg/L)	95% FL (mg/L)	RR
Western	Pyridaben	SUS	200	4.16 ± 0.68	0.93 (3)	4.96	4.02–5.70	1
		KK	200	4.25 ± 0.67	0.69 (3)	5.6	4.68–6.35	1.13
		VH	200	3.56 ± 0.63	2.29 (3)	5.32	4.23–6.18	1.07
		BS	200	4.01 ± 0.63	0.25 (3)	5.95	5.05–6.72	1.2
		BN	200	4.02 ± 0.65	2.09 (3)	5.71	4.74–6.49	1.15
Central		MR	200	3.66 ± 0.62	0.12 (3)	6.47	5.48–7.36	1.3
		KA	200	4.10 ± 0.64	0.71 (3)	6.44	5.55–7.24	1.3
Eastern		EB	200	4.21 ± 0.69	1.37 (3)	6.16	5.19–6.98	1.24
		TN	200	4.49 ± 0.72	0.97 (3)	6.17	5.22–6.95	1.24
Nyanza		KS	200	4.71 ± 0.72	2.49 (3)	5.57	4.69–6.28	1.12
		SA	200	4.20 ± 0.69	1.81 (3)	5.33	4.35–6.11	1.07
Rift valley		UG	200	3.76 ± 0.63	0.49 (3)	6.82	5.86–7.72	1.38
		TZ	200	4.06 ± 0.73	0.04 (3)	6.96	5.81–7.95	1.4
		NN	200	4.13 ± 0.65	0.58 (3)	6.75	5.85–7.57	1.36

Western	Abamectin	SUS	200	2.94 ± 0.48	0.74 (3)	3089.31	2269.00–3782.95	1
		KK	200	2.76 ± 0.51	1.43 (3)	4246.42	3134.31–5198.31	1.37
		VH	200	2.96 ± 0.46	0.09 (3)	4168.13	3356.92–4916.31	1.35
		BS	200	2.64 ± 0.47	0.64 (3)	4319.99	3292.52–5244.81	1.4
		BN	200	2.69 ± 0.44	1.08 (3)	4325.42	3444.55–5155.33	1.4
Central		MR	200	2.75 ± 0.46	0.08 (3)	4767.97	3850.98–5667.42	1.54
		KA	200	2.44 ± 0.45	0.68 (3)	4581.29	3493.61–5612.33	1.48
Eastern		EB	200	2.91 ± 0.51	0.2 (3)	4396.5	3352.09–5310.22	1.42
		TN	200	2.93 ± 0.51	1.52 (3)	4350.45	3340.59–5229.24	1.41
Nyanza		KS	200	2.88 ± 0.45	0.24 (3)	4201.76	3372.33–4969.76	1.36
		SA	200	2.75 ± 0.45	2.43 (3)	4100.59	3230.84–4894.78	1.33
Rift valley		UG	200	2.49 ± 0.49	0.73 (3)	5105.51	3910.34–6270.82	1.65
		TZ	200	2.58 ± 0.46	0.17 (3)	5359.01	4346.40–6453.85	1.73
		NN	200	2.99 ± 0.55	0.86 (3)	5040.48	3930.81–6043.45	1.63

Western	Imidacloprid	SUS	200	4.11 ± 0.69	0.18 (3)	955.68	761.82–1103.07	1
		KK	200	3.94 ± 0.68	0.36 (3)	1397.14	1188.75–1587.29	1.46
		VH	200	3.49 ± 0.61	1.94 (3)	1288.41	1082.51–1470.58	1.35
		BS	200	3.6 ± 0.68	0.37 (3)	1476.64	1241.16–1701.55	1.55
		BN	200	3.00 ± 0.60	0.27 (3)	1427.66	1187.70–1667.36	1.49
Central		MR	200	3.91 ± 0.81	0.51 (3)	1654.45	1388.68–1916.66	1.73
		KA	200	4.13 ± 0.79	0.61 (3)	1684.33	1447.96–1933.26	1.76
Eastern		EB	200	3.91 ± 0.77	1.65 (3)	1544.96	1296.75–1772.16	1.61
		TN	200	3.36 ± 0.72	0.63 (3)	1565.02	1276.63–1847.47	1.64
Nyanza		KS	200	4.16 ± 0.67	0.28 (3)	1230.25	1038.0–1392.80	1.29
		SA	200	4.14 ± 0.70	0.10 (3)	1168.39	960.90–1336.15	1.22
Rift valley		UG	200	3.55 ± 0.70	1.60 (3)	1726.1	1481.32–2026.78	1.81
		TZ	200	4.04 ± 0.86	0.21 (3)	1748.02	1480.99–2027.77	1.83
		NN	200	3.64 ± 0.79	0.79 (3)	1696.73	1411.02–1996.02	1.78

**Table 5:** Susceptibility of field populations of *Spodoptera frugiperda* to spinosad, spinetoram, and lufenuron.

Region	Insecticides	*P*	*N*	Slope ± SE	*χ * ^2^ (d*f*)	LC_50_ (mg/L)	95% FL (mg/L)	RR
Western	Spinosad	SUS	200	1.93 ± 0.28	0.1 (3)	0.26	0.19–0.34	1
		KK	200	1.47 ± 0.26	0.48 (3)	0.4	0.26–0.56	1.54
		VH	200	1.22 ± 0.24	1.84 (3)	0.39	0.23–0.58	1.5
		BS	200	1.53 ± 0.25	2.41 (3)	0.39	0.27–0.53	1.5
		BN	200	1.37 ± 0.25	2.47 (3)	0.39	0.24–0.56	1.5
Central		MR	200	1.47 ± 0.25	2.47 (3)	0.45	0.31–0.63	1.73
		KA	200	1.65 ± 0.27	0.25 (3)	0.43	0.29–0.58	1.65
Eastern		EB	200	1.22 ± 0.25	1.45 (3)	0.44	0.26–0.67	1.69
		TN	200	1.56 ± 0.26	0.76 (3)	0.44	0.30–0.62	1.69
Nyanza		KS	200	1.66 ± 0.27	2.99 (3)	0.37	0.25–0.51	1.42
		SA	200	1.50 ± 0.26	1.27 (3)	0.36	0.24–0.50	1.38
Rift valley		UG	200	1.51 ± 0.26	1.84 (3)	0.63	0.44–0.90	2.42
		TZ	200	1.28 ± 0.25	0.7 (3)	0.76	0.52–1.19	2.92
		NN	200	1.25 ± 0.24	0.75 (3)	0.61	0.41–0.93	2.35

Western	Spinetoram	SUS	200	1.86 ± 0.28	0.85 (3)	0.14	0.10–0.19	1
		KK	200	1.64 ± 0.26	1.20 (3)	0.18	0.12–0.24	1.29
		VH	200	1.64 ± 0.27	1.86 (3)	0.16	0.11–0.22	1.14
		BS	200	1.69 ± 0.26	0.95 (3)	0.19	0.14–0.25	1.36
		BN	200	1.63 ± 0.27	2.13 (3)	0.16	0.11–0.23	1.14
Central		MR	200	0.92 ± 0.23	1.33 (3)	0.27	0.16–0.48	1.93
		KA	200	1.19 ± 0.24	1.93 (3)	0.28	0.18–0.44	2
Eastern		EB	200	1.42 ± 0.27	0.13 (3)	0.25	0.16–0.37	1.79
		TN	200	1.41 ± 0.26	2.08 (3)	0.22	0.14–0.32	1.57
Nyanza		KS	200	1.85 ± 0.27	1.35 (3)	0.16	0.11–0.21	1.14
		SA	200	1.82 ± 0.29	0.72 (3)	0.16	0.11–0.22	1.14
Rift valley		UG	200	1.35 ± 0.28	0.07 (3)	0.35	0.22–0.55	2.5
		TZ	200	1.30 ± 0.25	2.09 (3)	0.48	0.33–0.80	3.43
		NN	200	2.03 ± 0.30	0.97 (3)	0.3	0.23–0.40	2.14

Western	Lufenuron	SUS	200	2.30 ± 0.43	0.50 (3)	2.82	1.87–3.59	1
		KK	200	3.00 ± 0.58	1.83 (3)	4.4	3.22–5.36	1.56
		VH	200	2.64 ± 0.47	0.64 (3)	4.32	3.29–5.25	1.53
		BS	200	2.34 ± 0.45	0.21 (3)	4.42	3.30–5.46	1.57
		BN	200	2.68 ± 0.46	0.83 (3)	4.45	3.46–5.36	1.58
Central		MR	200	2.55 ± 0.50	1.45 (3)	4.91	3.72–6.02	1.74
		KA	200	2.93 ± 0.58	1.01 (3)	4.88	3.65–5.92	1.73
Eastern		EB	200	3.21 ± 0.61	1.03 (3)	4.62	3.46–5.56	1.64
		TN	200	3.15 ± 0.49	2.49 (3)	4.62	3.77–5.41	1.64
Nyanza		KS	200	3.24 ± 0.56	0.69 (3)	4.25	3.26–5.09	1.51
		SA	200	2.78 ± 0.45	1.91 (3)	4.23	3.34–5.04	1.5
Rift valley		UG	200	2.51 ± 0.51	0.40 (3)	6.13	4.86–7.62	2.17
		TZ	200	2.37 ± 0.47	1.55 (3)	5.76	4.58–7.13	2.04
		NN	200	2.51 ± 0.51	2.67 (3)	5.05	3.80–6.22	1.79

**Table 6:** Pairwise correlation analysis of the LC_50_ values for nine insecticides in the 13 field populations of *Spodoptera frugiperda.*

	Pyridaben	Abamectin	Imidacloprid	Lambda-cyhalothrin	Deltamethrin	Indoxacarb	Spinosad	Spinetoram
Abamectin	0.907^*∗∗*^							
Imidacloprid	0.951^*∗∗*^	0.923^*∗∗*^						
Lambda-cyhalothrin	0.46	0.432	0.384					
Deltamethrin	0.940^*∗∗*^	0.982^*∗∗*^	0.931^*∗∗*^	0.445				
Indoxacarb	0.973^*∗∗*^	0.952^*∗∗*^	0.949^*∗∗*^	0.441	0.973^*∗∗*^			
Spinosad	0.907^*∗∗*^	0.936^*∗∗*^	0.854^*∗∗*^	0.389	0.935^*∗∗*^	0.947^*∗∗*^		
Spinetoram	0.943^*∗∗*^	0.840^*∗∗*^	0.848^*∗∗*^	0.4	0.890^*∗∗*^	0.939^*∗∗*^	0.922^*∗∗*^	
Lufenuron	0.878^*∗∗*^	0.976^*∗∗*^	0.907^*∗∗*^	0.419	0.959^*∗∗*^	0.944^*∗∗*^	0.912^*∗∗*^	0.818^*∗∗*^

## Data Availability

The data used and analyzed during the current study are available from the corresponding author upon request.
